# COVID-19 and MPXV: Twindemic Response and Dual Infections in Individuals in a US Metro

**DOI:** 10.3390/epidemiologia7010003

**Published:** 2025-12-24

**Authors:** Atiya Khan, Timothy A. Erickson, Louis Carrillo

**Affiliations:** 1 Houston Health Department, 8000 N Stadium Dr., Houston, TX 77054, USA; atiya.khan@houstontx.gov; 2 Laboratories for Emerging and Tropical Disease Research, Department of Epidemiology and Biostatistics, School of Public Health, Texas A&M University, 212 Adriance Lab Rd, College Station, TX 77843, USA; timerickson@exchange.tamu.edu

**Keywords:** COVID-19, MPXV, coinfection, surveillance

## Abstract

Background/Objectives: The purpose of this study was to identify shared and differing characteristics of individuals testing for both SARS-CoV-2 and MPXV in 2022 in the greater Houston metro area. Methods: Data from the Houston Electronic Disease Surveillance System (HEDSS) identified 7,754,198 SARS-CoV-2 PCR lab results and 1246 MPXVV PCR lab results in 2022. Three cohorts for analysis were created where tests were performed, as follows: those positive for both viruses, those negative for COVID-19 but positive for MPXV, and those positive for COVID-19 but negative for MPXV. Results: We identified 88 individuals positive for both viral infections, those negative for COVID-19 but positive for MPXV (*n* = 38), and those positive for COVID-19 but negative for MPXV (*n* = 96). While groups were generally similar in regard to demographics (age, sex, and race) and risk factors reported, key differences in timing of testing and risk factors were reported. Notably, there was statistically significant difference in the time between *t*-tests for dual-infected individuals (99 days) compared to MPXV-positive only (58 days, *p* < 0.01) or COVID-19 positive only (63 days, *p* < 0.01). Conclusions: In the setting of multiple disease outbreaks, the characteristics of infected patients may be largely similar. Some people with dual infection may show unusual test results or symptom patterns compared with those with only one infection. Large public health studies with robust reporting systems and laboratory screening are vital for early detection of dual infections. Public health strategies to educate providers and outreach teams enhance response during concurrent outbreaks. Further research is needed on behavior and risk factors in communities with simultaneous outbreaks.

## 1. Introduction

The first cases of COVID-19 in Houston, Texas (United States of America) were identified in 2020 [1]. During the subsequent COVID-19 pandemic, a widespread MPXV outbreak also occurred in 2022 [2]. The MPXV outbreak, which occurred before the world had fully controlled COVID-19 added a further burden to public health systems. MPXV, previously known as monkeypox, is a viral illness caused by the monkeypox virus, a species of the genus Orthopoxvirus. In 2022, a global outbreak of MPXV was caused by the clade IIb strain [3]. Additionally, with the public health and economic crisis caused by COVID-19 still ongoing, the MPXV outbreak exacerbated the burden on health care systems and lead to “combination virus” hazards [4,5,6]. Few studies have employed broad public health data to assess emerging risks of MPXV in the context of the COVID-19 pandemic so as to guide countries in developing response strategies [6,7,8,9,10,11].

The Houston Health Department (HHD) is an agency that conducts disease surveillance in the city of Houston, Texas. The department is responsible for approximately 4.86 million people in the city and for investigating more than 80 infectious diseases in Harris, Montgomery, and Fort Bend Counties [12].

### History and Overview

Infections with both SARS-CoV-2 and MPXV can occur alongside coinfections with bacteria, fungi, and other viruses [7,8,9,13,14,15,16,17]. MPXV in particular, is well known for coinfections with HIV, as well as other sexually transmitted infections [18,19,20,21]. COVID-19 patients are at risk of various complications, and those with co-morbidities and associated infections are particularly vulnerable [22,23]. There is some overlap in the clinical presentation of COVID-19 and MPXV. The symptoms most frequently associated with MPXV are dermatologic, including papules, vesicles, and scarring [19,24]. While most well known for respiratory symptoms, COVID-19 may also demonstrate manifestations such as an erythematous maculopapular rash, erythema multiforme, vesicular rash, vascular livedo reticularis, figurate erythema, or a flexural rash [25]. While the possibility of dual infection with SARS-CoV-2 and MPXV exists, this phenomenon has not been well studied, and large studies of concurrent disease reporting of both COVID-19 and MPXV are lacking [22,26,27,28].

The purpose of this study is to identify individuals with both SARS-CoV-2 and MPXV tests in 2022 and characterize observed risk factors and symptoms in those who were infected with both viruses when compared to those infected with only a single virus. To address these key gaps in knowledge, analytical surveillance methods have been developed to match individuals with both diseases. Benefits of early and dual surveillance include identifying potential risk factors encouraging healthcare systems to respond more effectively and efficiently, possible prevention of complications, and reducing the overall cost of care.

## 2. Materials and Methods

During the time period of the study (2022), the disease burden for both COVID-19 and MPXV in Houston was assessed by analyzing PCR test results. Laboratories in the jurisdiction of HHD generate digital reports of PCR test results in the form of electronic laboratory reporting (ELR), which are then securely transmitted to the HHD using standardized formats. Upon receipt, the data undergoes validation for completeness and accuracy, and the information is integrated into the Houston Electronic Disease Surveillance System (HEDSS), a Maven-based surveillance system, developed by CONDUENT. ELR automates the reporting process by translating information into an electronic message that can be automatically sent and processed. Continuous monitoring ensures that the database remains updated with the latest PCR test results, allowing for effective public health monitoring and response. Self-reported demographic information was obtained during case investigation, scheduling of the test at designated sites, or from patient records. Laboratory information and epidemiological case investigation data was extracted for investigations of either COVID-19 and/or MPXV. Our analysis included only individuals tested in 2022. For the purposes of this study, antigen testing alone was not considered sufficient for identification of a COVID-19 case. Our health department uses PCR test and not antigen testing to define the case.

### 2.1. Description of COVID-19 Outbreak in Houston in 2022

For the purposes of this study, cases of COVID-19 were defined as persons with a PCR test for SARS-CoV-2. In the year 2022, the Houston Health Department received 7,754,198 PCR test results. Of these, 1,489,336 (19%) had positive lab results.

### 2.2. Description of MPXV Outbreak 2022

For the purposes of this study, cases of MPXV were also defined as persons with a PCR test for MPXV. In the year 2022, the Houston Health Department received 3639 lab tests for MPXV. Of these, 1246 (34%) were positive.

### 2.3. Creation of Dataset and Statistical Analysis

The datasets were cross-referenced, and matches were identified, using the proq sql function in SAS 9.4 with variables first name, last name, and date of birth. Statistical analysis of data was performed in Statistical Analysis System (SAS, SAS Institute, Cary, NC, USA) 9.4. Three study groups were created as follows: (1) those who were positive for SARS-CoV-2 and MPXV, (2) those who were positive for only SARS-CoV-2 and negative for MPXV, and (3) those who were negative for SARS-CoV-2 and positive for MPXV. Statistical comparisons were conducted using Pearson’s Chi square testing, with Fisher’s exact test used for instances where a given cell had <5 observations. Student’s *t* test was employed to compare continuous variables where 30 or more observations were present in each comparison group. These statistical comparisons were conducted using Stata version 14.2 (Statacorp, College Station, TX, USA).

### 2.4. Variables of Interest

Information regarding demographics, symptomatology, risk factors, and data on temporality of infections were identified for those cases identified in each of the three study groups. When multiple test dates for SARS-CoV-2 were available, we selected the test date that was most proximal to the date of MPXV testing. Temporality of testing was examined in both the initial continuous and in a categorized format (five categories, 0–7 days, 7–14 days, 14–60 days, 60–90 days, and more than 90 days). Demographic variables extracted for this study included gender, race and ethnicity. Data on symptoms primarily included rash and lymphadenopathy, immunocompromised status, and HIV status. Additional risk factors examined as a part of routine surveillance and interview included if patients were healthcare workers exposed to MPXV at work, if they received smallpox vaccination prior to infection, patient hospitalization, death caused by illness, potential contact, exposure, and travel history.

The duration between COVID-19 infection and MPXV infection in all the three cohorts was studied individually using the variable ‘specimen collection date’. We defined delayed testing as more than one week between SARS-CoV-2 and MPXV specimen collection. It roughly aligns with the typical incubation period of both viruses.

## 3. Results

We identified a total of 88 unique individuals infected with both COVID-19 and MPXV in 2022 (these comprised the first study group). We also identified 38 unique individuals negative for SARS-CoV-2 and positive for MPXV (the second group). Finally, we also identified a total of 96 SARS-CoV-2 positive unique individuals and MPXV negative (the third and final group).

### 3.1. Demographic Investigation

The cohort positive for both SARS-CoV-2 and MPXV had 88 patients in total with a mean age of 38 years, which was generally similar to ages for either the SARS-CoV-2 negative (−) MPXV positive (+) (mean age 38 years) or SARS-CoV-2+ MPXV− (37 years); no statistically significant differences were noted. All cohorts were predominantly male, though differences in the proportion of males in the SARS-CoV-2+ MPXV+ and each of the other two groups were noted. When those who were SARS-CoV-2+ and MPXV+ during 2022 (97% male) were compared to those in the SARS-CoV-2+ MPXV− group (70% male), a statistically significant difference (*p* < 0.0001, OR = 11.7, 95% CI = 3.3–61.8) was noted. A similar difference was noted when comparing the single infection groups to each other (no meaningful OR reported as 100% of one comparison group was male resulting in a cell with a value of 0). No differences in the distributions of race were observed between the groups, with comparisons of white race between those with SARS-CoV-2+ MPXV+ (41%) and SARS-CoV-2− MPXV+ (34%, *p* = 0.48, OR = 1.33, 95% CI = 0.6–3.2) or SARS-CoV-2+ MPXV− (32%, *p* = 0.22, OR = 1.45, 95% CI 0.8–2.8) both lacking conventionally statistically significant values. The same held for black race, where comparisons of SARS-CoV-2+ MPXV+ (32%) and SARS-CoV-2− MPXV+ (42%, *p* = 0.26, OR = 0.64, 95% CI = 0.3–1.5) or SARS-CoV-2+ MPXV negative (32%, *p* = 0.96, OR = 0.97, 95% CI 0.5–1.9) both lacked conventionally statistically significant values (Table 1).

### 3.2. Risk Factors and Symptomatology

There were no statistically significant differences between groups in risk factors except for reported smallpox vaccination prior to MPXV testing (38% in the SARS-CoV-2+ and MPXV+ group vs. 7% in the SARS-CoV-2− MPXV+ group, OR = 8.5, *p* = 0.02, 95% CI 1–385). Persons with only SARS-CoV-2+ were not interviewed for intention to receive or reception of smallpox vaccination. There was no difference in the number immunocompromised when comparing either those positive for both agents vs. those with only MPXV+ (56% vs. 64%, OR = 0.71, 95% CI = 0.2–2.0) or those with only SARS-CoV-2+ (56% vs. 33%, OR = 2.6, *p* = 0.4, 95% CI = 0.3–29). Immune compromise status was not available for the vast majority of SARS-CoV-2+ MPXV− persons. Only a single fatality was recorded in any of the study groups (in the SARS-CoV-2+ and MPXV+ group) (Table 2), although the cause of death was not related to either infection. There was also no difference in rash symptomatology in those in the SARS-CoV-2+ and MPXV+ group when compared to those in the MPXV+ only group (96% vs. 92%, mean = 95.94,932.3, OR = 2.0, *p* = 0.6, 95% CI 0.16–18.2). Of those who had data available, a total of 19 (41%) had swollen lymph nodes in the SARS-CoV-2+ MPXV+ group, while the SARS-CoV-2-MPXV+ and SARS-CoV-2+MPXV− had 10 (56%) and 2 (40%) individuals with reported swollen lymph nodes, respectively; these differences were not statistically significant.

### 3.3. Temporal Investigation

**Figure 1:**
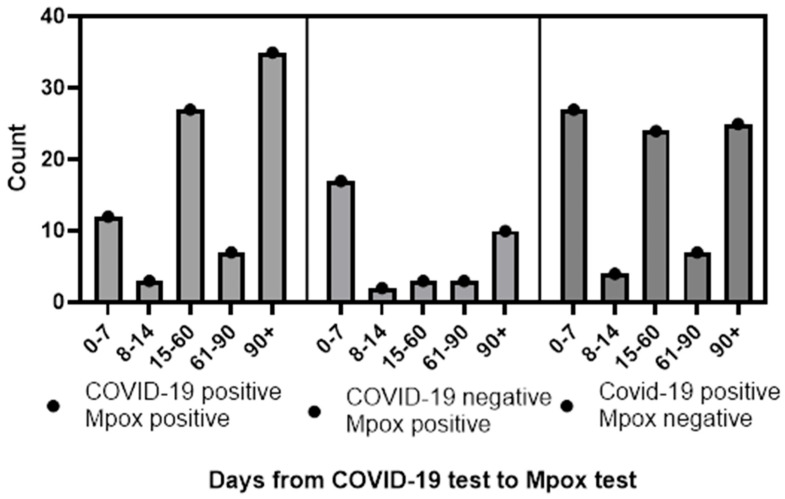
Temporality of SARS-CoV-2 and MPXV infections in Houston, Texas, by days from SARS-CoV-2 test to MPXV test.

Data on temporality of testing were available for nearly all of the observations (a single MPXV+ and SARS-CoV-2+ case was missing data on test timing). Of these, the majority (205/221, 93%) were tested first for SARS-CoV-2 and then for MPXV. A total of 56 (27%) of these were tested for both diseases within a week of each other. When analyzing only those with a SARS-CoV-2 test first, some differences were observed in the timing of positivity for disease between groups. Those with SARS-CoV-2+ and MPXV+ tests had a mean of 99 days between tests and SD 87.6, while those with SARS-CoV-2− and MPXV+ tests had a mean of 58 days between tests and SD 74.77. This difference was statistically significantly longer when tested as a continuous variable (*p* < 0.01). When we analyzed the data dichotomously (as those with delayed test positivity vs. those without) we also observed a difference. This difference was also statistically significant (OR = 0.2, 95% CI = 0.1–0.5, *p* < 0.01, Figure 1). For those who were tested for both viruses within a week, a total of 12 (14%) tested positive for SARS-CoV-2 and MPXV within a week, while 17 (49%) tested positive for MPXV after a negative SARS-CoV-2 test.

Similar observations were made when we compared the SARS-CoV-2+ MPXV+ group to the SARS-CoV-2+ MPXV− group (mean 99 days between tests vs. mean 63, statistically significant difference tested continuously at the *p* < 0.01 level, and dichotomously different with OR = 0.4, 95% CI = 0.2–0.8, *p* < 0.01 level).

There were no statistically significant differences, continuously or dichotomously, between the SARS-CoV-2+ MPXV− and SARS-CoV-2− MPXV+ groups, though the *p* value for the dichotomous comparison of 0.07 did approach significance.

## 4. Discussion

Our study uses a large public health approach. To date, very few studies have examined dual infections or documented a recent history of both COVID-19 and MPXV in the same individuals. None have included a comparable number of people across all three possible infection-combination groups. Although previous reports have described dual infections, they are almost entirely based on small, hospital-based cohorts and are typically limited to patients with more severe disease [22,26,27,28]. Data drawn from ELR reporting to a local health department have the potential to capture relatively large numbers in a twindemic response that exists in a way hospital-based case studies cannot. We leveraged this capacity to provide the relatively large data presented in this study.

Our results are generally reflective of the expected characteristics age, race, and gender of either MPXV of COVID-19 infections based on previously published data. Those with MPXV only were largely 30–50-year-old males with HIV, a history of intimate contact, and presenting with a history of rash [19]. While the prevalence of rash in those with COVID-19 seems higher than that found in other studies, it should be noted that COVID-19 cases were only included if they also had a history of MPXV testing; given that rash is a hallmark of MPXV infection, it is likely this finding is the result of a selection bias [29]. The same explanation likely applies to the higher-than-expected number of males in the COVID-19 only group. Studies on cohorts with HIV status information have reported that 40% of MPXV cases had HIV dual-infection [19,21,30]. In general, the groups appear the same in most respects, which we hypothesize is related to testing expectations for MPXV infections.

There are, however, several differences worth noting. The first is the differential temporality clearly observed in this study. Persons infected with both COVID-19 and MPXV had a mean of 98 days between the positive SARS-CoV-2 test and the subsequent positive MPXV test, while persons with positive or negative SARS-CoV-2 tests had nearly a month less between their respective MPXV tests. No immediately clear explanation is available. It is possible that these differences reflect a behavioral factor, notably that a person with a positive SARS-CoV-2 test may choose to isolate and thus reduce exposure possibility to MPXV. There are a number of problems with this theory, however. First, the time between testing for those with negative SARS-CoV-2 tests and positive MPXV tests and those with positive SARS-CoV-2 tests and negative MPXV tests are similar, which seems to suggest no such behavioral factor is in play. Second, while the compliance with isolation protocols for COVID-19 was never perfect in the United States, it seems likely that this compliance would be especially low by 2022 (when the data from this study originated), and thus unlikely to broadly effect the timing of these tests [31]. It is also possible this delay is the result of cross protection from viral interference induced by the SARS-CoV-2 infection. This could take the form of cross protection via antibodies or a general upregulation of innate immune system components. However, limited published evidence supports these hypotheses.

The difference in reported uptake of smallpox vaccination prior to MPXV infection is equally difficult to explain. That the SARS-CoV-2+MPXV+ population would be eight times more likely to have been vaccinated than the SARS-CoV-2-MPXV+ population seems to be opposed to any behavioral theories, and there are no relevant biological theories we are aware of that could explain this difference. Reporting bias or incomplete vaccination records are possible contributors, especially because our team does not have access to the Texas ImmTrac2 immunization registry and vaccination status often relies on self-report. Dual-positive individuals may also have more frequent healthcare contact, which can increase both vaccination opportunities and documentation.

A significant breakthrough in this work is the application of data analytics methods that provide reliable insights into transmission of concurrent infections and thereby guiding surveillance strategies and healthcare preparedness. Another important aspect to consider is the overlap in clinical symptoms between the two infections, as most MPXV patients present with fever, which is also common among COVID-19 patients. Overlap in clinical symptoms requires careful evaluation to avoid misclassification as they may create difficult situations in the setting of concurrent outbreaks.

Direct costs of MPXV illness include evaluation, treatment, and medication, while indirect costs involve work absenteeism and mortality; however, consistent cost estimates remain [32]. Hospitalization for COVID-19 has averaged more than $10,000 per patient in the U.S. [33,34]. Because these costs have increased over time, understanding how to reduce unnecessary clinical visits, need for multiple tests, and resource use is important to reduce strain on healthcare facilities and reduced expenses for patients.

There have been relatively few instances of concurrent epidemics on this scale in the modern era, and response strategies might need to be adapted or modified in such scenarios. One such component is screening to establish early diagnosis of cases, monitor spread, and overall surveillance and being aware of the possibility of dual infections and testing for both including individuals who already tested for one. Additionally, investigators should be aware of recent infections that may influence behavior and other risk factors, when evaluating need to test for other concurrent infections and community outbreaks. This is especially beneficial to vulnerable populations that have financial challenges for multiple visits as well as those who cannot commute and are physically isolated.

Our study has several limitations. Foremost, some epidemiologic variables contained substantial missing data, because the data were drawn primarily from electronic laboratory reporting (ELR) during an outbreak response, and not from electronic case reports (ECR). Our ELR does not capture details about clinical and exposure information like ECR. Follow-up was also challenging, particularly among individuals who were lost to follow-up or among populations at higher risk, including those experiencing homelessness, which further contributed to missing or incomplete data. Some interviews were dependent on people to recall which is a limitation of ELR study. Additionally, there are differences in case investigations between the two diseases: they are transmitted in different ways, have different risk factors, and involve different monitoring periods. While employing the resources of a large health department was vital in allowing us to identify cases of each infection, individually or as multiple infections, the emphasis of the health department on conducting investigations of these diseases as separate entities limits the depth of our study. This limitation prevented analysis of certain variables (for example, in depth rash analysis). Even with the large pool of data drawn from the Health Department, the numbers available for those infected with both MPXV and COVID-19 were somewhat limited. Although multiple *t*-tests were helpful to streamline the analysis, incorporating ANOVA in future studies would enhance interpretability and provide an additional layer of statistical robustness.

## 5. Conclusions

To our knowledge, this is the first study to analyze dual outbreaks of COVID-19 and MPXV using large public health datasets. This analysis identified that individuals with dual infections existed, that at times there were differences in laboratory test time points, and that utilizing the surveillance system can identify those scenarios during concurrent outbreaks. Efforts should focus on using resources more efficiently, improving outbreak response capacity and ensuring teams can manage testing, supplies, and vaccinations. Integrated multi-disease testing, better provider education and stronger coordination across response teams will support a more effective and holistic response.

## Figures and Tables

**Table 1:** Demographic characteristics of patients with SARS-CoV-2 and MPXV tests.

	SARS-CoV-2 and MPXV Positive (*n* = 88)	SARS-CoV-2 Negative MPXV Positive (*n* = 38)	SARS-CoV-2 Positive MPXV Negative (*n* = 96)
Median Age (Range)	38.17 (SD = 12.1)	37.86 (SD = 10.2)	36.98 (SD = 19.2)
Male Sex (%)	85/88 (96.6%)	38/38 (100%)	68/96 (70.8%)
Race			
White (%)	36/88 (40.9%)	13/38 (34.2%)	31/96 (32.3%)
Black (%)	28/88 (31.8%)	16/38 (42.1%)	31/96 (32.3%)
American-Indian/Alaskan native (%)	<5	<5	<5
Asian (%)	<5	<5	5/96 (5.2%)
Other (%)	8/88 (9.1%)	<5	9/96 (9.4%)
Unknown (%)	12/88 (13.6%)	<5	19/96 (19.8%)

**Table 2:** Risk factors and symptomatology for patients with SARS-CoV-2 and MPXV tests.

Risk Factors Symptoms	SARS-CoV-2 and MPXV Positive (*n* = 88)	SARS-CoV-2 Negative MPXV Positive (*n* = 38)	SARS-CoV-2 Positive MPXV Negative (*n* = 96)
Confirmed HIV Status	34/61 (55.73%)	16/25 (64%)	*
Immunocompromised	34/61 (55.73%)	16/25 (64%)	<5
Intimate Contact	49/59 (83.05%)	16/23 (69.56%)	*
Noted Health Conditions	14/45 (31.11%)	<5	3/3
Smallpox Vaccination	14/37 (37.83%)	<5	*
Hospitalized	<5	<5	0
Fatality	<5	0	*
Travel Prior to Contact	12/51 (23.52%)	<5	*
Rash	71/74 (95.94%)	24/26 (92.3%)	15/18 (83.33%)
Swollen Lymph Node	19/46 (41.30%)	10/18 (55.55%)	<5

* = Persons MPXV negative but COVID-19 positive are not investigated for these variables and as such they are missing from this table.

## Data Availability

The original contributions presented in this study are included in the article. Further data is available upon formal request at the office of legal affairs.
